# Gamma knife for the treatment of choroidal breast cancer metastasis causing retinal detachment: A case report

**DOI:** 10.3389/fonc.2025.1647524

**Published:** 2025-11-25

**Authors:** Jee Yan Ong, Ereena Alfian, Ramesh Kumar, Fuad Ismail, Niki Wai Wye Ho, Mae-Lynn Catherine Bastion, Othmaliza Othman

**Affiliations:** 1 Department of Ophthalmology, Faculty of Medicine, Universiti Kebangsaan Malaysia, Kuala Lumpur, Malaysia; 2 Department of Neurosurgery, Faculty of Medicine, Universiti Kebangsaan Malaysia, Kuala Lumpur, Malaysia; 3 Department of Radiotherapy and Oncology, Faculty of Medicine, Universiti Kebangsaan Malaysia, Kuala Lumpur, Malaysia

**Keywords:** breast carcinoma, case report, choroidal metastasis, gamma knife radiosurgery, multidisciplinary care

## Abstract

**Introduction:**

Choroidal metastasis generally has poor prognosis and most commonly originates from breast carcinomas. While systemic chemotherapy offers therapeutic benefits, local therapies are often necessary for symptom management and tumor control. Gamma knife radiosurgery (GKR), which was originally developed for intracranial lesions, has emerged as a promising treatment for choroidal metastasis, offering high precision and minimized toxicity with fewer ocular side effects compared with conventional radiotherapy. This case report explores the use of GKR in a patient with choroidal and brain metastases from breast carcinoma.

**Case presentation:**

A 44-year-old woman with a history of treated left breast carcinoma presented with 3 months’ gradual vision loss in her left eye. Her visual acuity at presentation was counting fingers (CF). Imaging revealed a choroidal metastasis along with multiple brain metastases. The patient underwent GKR for both choroidal and intracranial metastases, receiving doses ranging from 16 to 18 Gy at 50%–90% isodose. Following treatment, significant tumor regression was observed, with a marked reduction in retinal detachment and vision improvement to 6/18. At 6 months post-GKR, both the choroidal mass and the retinal detachment had fully resolved; however, her visual acuity remained limited due to foveal atrophy.

**Conclusion:**

This case demonstrates the potential of GKR as a noninvasive and effective modality for the simultaneous treatment of choroidal and intracranial metastases. In palliative settings, especially for patients with limited life expectancy, GKR can provide symptomatic relief and improved quality of life with minimal invasiveness, which is particularly valuable for younger patients facing advanced metastatic cancer. The importance of a multidisciplinary approach in the management of complex metastatic diseases is also highlighted. Future studies are warranted to fully define the role of GKR in choroidal metastasis and its long-term sequelae.

## Introduction

Choroidal metastasis is the most common form of intraocular malignancy in adults, with breast carcinomas being the most frequent primary source, accounting for 40%–53% of all choroidal metastasis cases (1–4). Given that the intraocular dissemination of tumor cells is largely via the hematogenous route, the rich vascular supply of the choroid makes it a preferred metastatic site, accounting for nearly 90% of all uveal metastases (1, 3). The prognosis for choroidal metastases is typically poor (1–3, 5). While systemic chemotherapy targeting the primary tumor can also be beneficial for choroidal lesions due to the highly fenestrated vasculature of the choroid allowing for good penetration of systemic agents, local therapies are often necessary to manage ocular symptoms such as eye pain, swelling, and vision loss, particularly in cases resistant to systemic therapy or with isolated ocular manifestation (1, 2). Over the years, local therapies have advanced from enucleation in the 1980s to globe-sparing techniques such as external beam radiation therapy (EBRT), brachytherapy, and proton beam radiotherapy, each demonstrating significant tumor regression. Gamma knife radiosurgery (GKR), which was originally developed for intracranial lesions, has also been adapted for orbital use, offering advantages in precision and reduced toxicity (6, 7). However, its use in the treatment of ocular metastasis remains underexplored due to the high cost of the equipment and the restricted accessibility to only major urban medical centers. Published data detailing the GKR technique and long-term outcomes are also limited. This paper reports on a case of breast carcinoma with choroidal and brain metastases treated with GKR.

## Case presentation

**Figure 1:**
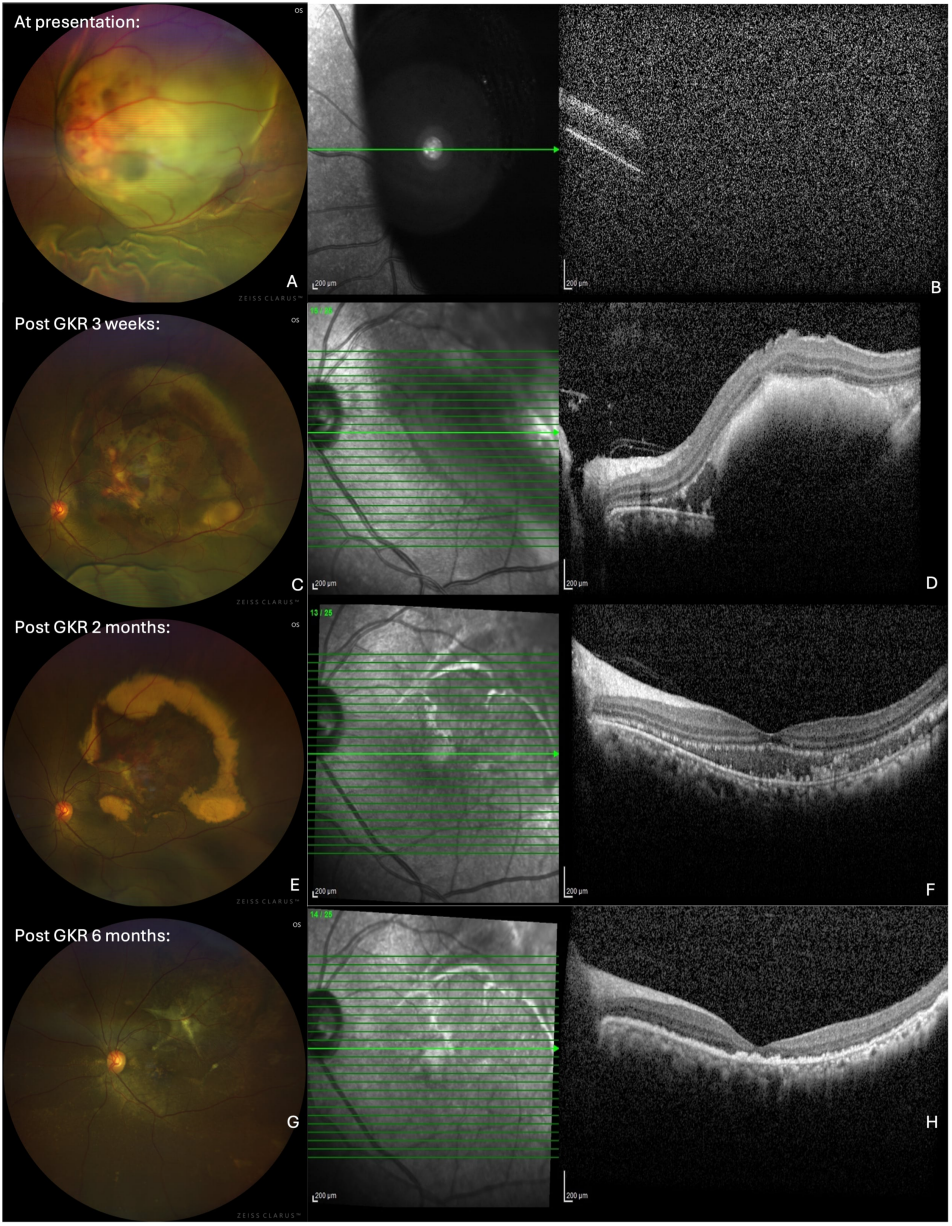
Wide-field fundus photographs (left column) and optical coherence tomography (OCT) imaging (right coloumn) showing left eye choroidal mass. **(A)** At presentation: subretinal mass in the papillomacular bundle, with associated subretinal haemorrhages and extensive serous detachment extending from 11 to 4 o’clock. **(B)** At presentation: OCT did not show useful information as the retinal lesion extended beyond the window capturable by the machine. **(C)** Post GKR 3 weeks: significant reduction in the size of the choroidal mass and exudative retinal detachment. **(D)** Post GKR 2 months: Further decrease in the size of subretinal convexity and adjacent subretinal fluid, with persistent hyperreflective foci in the outer retinal layers correlating with the hard exudates. **(E)** Post GKR 2 months: Reduced size of choroidal mass, with areas of hard exudations and residual subretinal hemorrhage. **(F)** Post GKR 2 months: Further decrease in the size of subretinal convexity and adjacent subretinal fluid, with persistent hyperreflective foci in the outer retinal layers correlating with the hard exudates. **(G)** Post GKR 6 months: Completely resolved choroidal mass and retinal detachment. **(H)** Post GKR 6 months: Resolution of the choroidal lesion and adjacent subretinal fluid, with areas of hyperreflective foci and irregularities observed at the RPE level, loss of ellipsoid zone and reduced subfoveal thickness.

A 44-year-old lady with a history of left breast carcinoma, previously treated with wide round block mammoplasty, chemotherapy, and radiotherapy, had no recurrences during her most recent annual follow-up with the breast surgeons. A year on, she presented to us with a 3-month history of gradual-onset blurry vision in her left eye, predominantly affecting the nasal visual field, with no eye pain or redness. On examination, her best-corrected visual acuity (BCVA) was counting fingers (CF) in the left eye, with a grade 3 positive relative afferent pupillary defect (RAPD), characterized by immediate pupillary dilation on the swinging flashlight test (8). The anterior segment examination was unremarkable, with an intraocular pressure (IOP) of 8 mmHg and no anterior chamber (AC) activity. Fundus examination revealed a subretinal mass in the papillomacular bundle, measuring 10-disc diameters, with associated subretinal hemorrhages and an extensive serous detachment extending from 11 to 4 o’clock; vitreous was otherwise clear (**Figure 1A**). The right eye examination was normal. The initial optical coherence tomography (OCT) imaging on presentation did not show useful information as the retinal lesion extended beyond the window capturable by the machine (**Figure 1B**).

**Figure 2:**
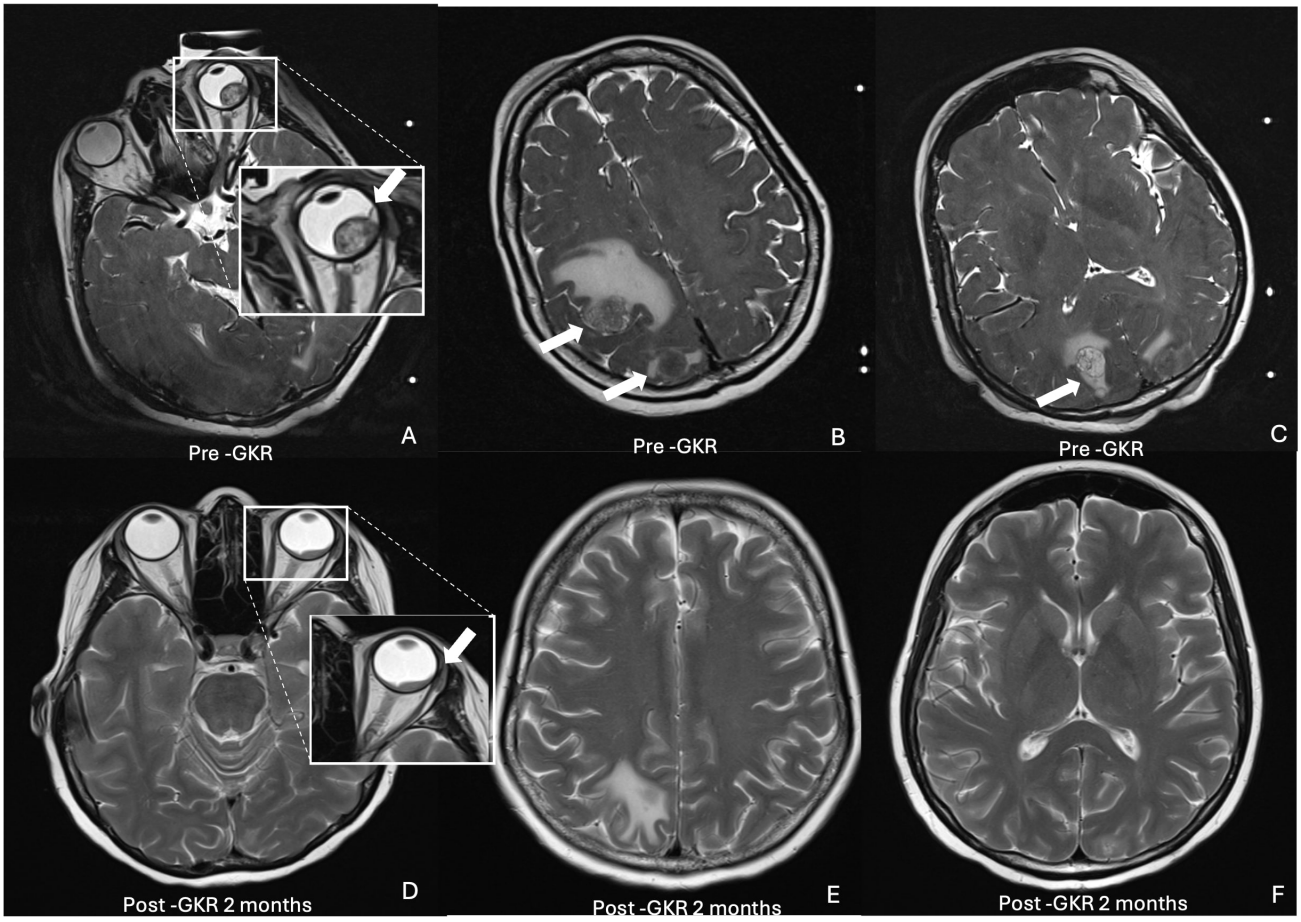
Axial T2-weighted MRI brain and orbit showing choroidal and multiple intracranial metastatic lesions pre (top row) and post GKR 2 months (bottom row). **(A)** inset. Intraocular mass (arrow) at superolateral left globe. **(B)** Metastatic nodules with prelesional oedema (arrows) at right parietal and occipital lobes **(C)** Metastatic nodule (arrow) at right occipital lobe. **(D)** inset. Reduced size of intraocular mass (arrow). **(E)** Reduced and resolved intracranial nodules at right parietal and occipital lobes. **(F)** Resolved nodules at occipital lobe.

An MRI scan of the brain and the orbit was arranged for the patient, which showed multiple enhancing metastatic nodules in the cerebral hemispheres and the right cerebellum, with the largest lesion measuring 1.9 cm × 1.7 cm × 2.2 cm (**Figures 2A–C**). An enhancing choroidal mass at the superolateral aspect of the left globe, measuring 1.1 cm × 0.7 cm × 1.4 cm, was also detected (**Figure 2A**). A staging PET scan was arranged, showing features of fluorodeoxyglucose (FDG)-avid disease in the brain, cervical spine, mediastinal nodes, mesenteric nodes, right lung, liver, spleen, stomach, muscle, and bone, indicating advanced metastatic disease.

The patient was co-managed under the ophthalmology, neurosurgery, general surgery, and oncology teams. Given the presence of both choroidal and brain metastases, she was planned for stereotactic GKR. Eye globe fixation was achieved medically using retrobulbar and periocular anesthesia to induce globe akinesia and mechanically with external fixation of four recti muscles and temporary tarsorrhaphy under the same surgical setting. The recti muscles were identified and externalized to the skin with a retinal band, followed by temporary tarsorrhaphy. Following globe fixation, the Leksell stereotactic head frame was applied to ensure head immobilization and precision of radiation during gamma knife delivery. A pre-GKR MRI was then performed to guide the tumor delineation and treatment planning. A treatment planning software was utilized for radiation and dose planning, sparing the critical ocular structures where possible. Gamma knife treatment was delivered to the left eye choroidal mass and the five largest intracranial lesions, followed by a top-up radiosurgery session a week later targeting the remaining 17 smaller intracranial lesions. The total GKR dose given to the lesions was 16–18 Gy at 50%–90% isodose. The procedures were completed in under 60 min, and the patient was discharged home the following day after both sessions.

At 3 weeks post-GKR, the patient’s BCVA showed a slight improvement from CF to 6/60. There was a significant reduction in the size of the choroidal mass and exudative retinal detachment on fundoscopy examination (**Figure 1C**). OCT now revealed a subretinal convexity with back shadowing and secondary retinal detachment with adjacent subretinal fluid containing hyperreflective foci (**Figure 1D**).

At 2 months after GKR, the patient’s BCVA in the left eye had improved to 6/18. The size of the choroidal mass had further reduced, with areas of hard exudations and residual subretinal hemorrhage (**Figure 1E**). A repeat OCT showed a further decrease in the size of the subretinal convexity and adjacent subretinal fluid, with persistent hyperreflective foci in the outer retinal layers correlating with the hard exudates observed on the fundus photo (**Figure 1F**). A follow-up MRI demonstrated a reduction in the size of the intracranial masses, with the resolution of several masses. The size of the enhancing choroidal mass was also significantly reduced from the previous 0.9 cm × 1.3 cm × 1.4 cm to 0.5 cm × 1.2 cm × 0.6 cm (**Figures 2D–F**).

At 6 months following treatment, her left eye BCVA was CF, although the choroidal mass and the retinal detachment had completely resolved (**Figure 1G**). OCT imaging showed resolution of the choroidal lesion and adjacent subretinal fluid, with the foveal contour retained. However, there were areas of hyperreflective foci and irregularities observed at the retinal pigment epithelium (RPE) level, along with the disruption of the ellipsoid zone. The reduced subfoveal thickness also indicated some degree of foveal atrophy (**Figure 1H**). **Table 1** summarizes the timeline of the case presentation.

**Table 1:** Case timeline.

Time point	Clinical event/investigation	Findings/management
1 year before presentation	Annual breast cancer follow-up	No recurrence detected
3 months before presentation	Onset of LE symptoms	LE gradual-onset painless blurry vision
At presentation	Ophthalmic exam	• LE BCVA: Counting fingers (CF). • RAPD: Grade 3 positive. • Fundus examination (**Figure 1A**): Subretinal mass (10 DD) in the papillomacular bundle with subretinal hemorrhages and serous RD (11 to 4 o’clock).
	OCT macula (**Figure 1B**)	Inconclusive due to lesion extent
	MRI brain and orbit (**Figures 2A–C**)	• Choroidal mass: 1.1 cm × 0.7 cm × 1.4 cm. • Multiple brain and cerebellar metastases.
GKR	GKR procedure	16–18 Gy at 50%–90% isodose to choroid + brain lesions in two sessions 1 week apart
3 weeks post-GKR	Ophthalmic exam	• LE BCVA: 6/60. • Fundus examination (**Figure 1C**): Significant reduction in mass and serous RD.
	OCT macula (**Figure 1D**)	Subretinal convexity and secondary retinal detachment, adjacent subretinal fluid containing hyperreflective foci. CST: non-recordable
2 months post-GKR	Ophthalmic exam	• LE BCVA: 6/18. • Fundus examination (**Figure 1E**): Further reduction in mass size, hard exudates, and subretinal fluid.
	OCT macula (**Figure 1F**)	Further decrease in subretinal convexity and fluid, persistent hyperreflective foci CST: 362 μm
	MRI brain and orbit (**Figures 2D–F**)	Reduced choroidal mass: 0.5 cm × 1.2 cm × 0.6 cm, reduction in brain metastases
6 months post-GKR	Ophthalmic exam	• LE BCVA: CF. • Fundus examination (**Figure 1G**): Resolution of choroidal mass and RD.
	OCT macula (**Figure 1H**)	Resolved subretinal convexity and fluid with intact foveal contour. Presence of hyperreflective foci and RPE irregularity, ellipsoid zone disruption, and reduced subfoveal thickness (foveal atrophy) CST: 132 μm

*LE BCVA*, left eye best-corrected visual acuity; *GKR*, gamma knife radiosurgery; *RAPD*, relative afferent pupillary defect; *RD*, retinal detachment; *OCT*, optical coherence tomography; *CST*, central subfield thickness.

## Discussion

According to the American Academy of Ophthalmology (AAO), the incidence of choroidal metastasis increases as the long-term survival rate of patients with cancer continue to improve over the years, reflecting a growing need for effective local therapies to optimize the visual outcomes and quality of life of these patients (9). Given the generally poor prognosis of ocular metastasis, which has an average survival rate of less than 1 year, its management remains largely symptomatic and palliative (5, 9). Systemic treatment is generally the first-line approach, with various options of local modalities available, which, while effective, come with each own set of challenges and limitations. Among these, radiotherapy has long played a central role in the local treatment of choroidal metastasis, with commonly employed methods including EBRT, plaque brachytherapy, proton beam therapy, and stereotactic options such as GKR.

The conventional EBRT is one of the most widely used due to its accessibility, noninvasive nature, cost-effectiveness, and its well-established tolerability, rendering it especially beneficial for use in patients with limited life expectancy (4). The tumor regression rate with EBRT has been reported to range between 85% and 93% (10–13), with visual rehabilitation achieved in 57%–89% of cases (3, 10). However, the broad radiation field and the relatively higher doses of EBRT (compared with other localized options) can damage the neighboring radiosensitive intraocular structures, with complications such as cataracts, radiation retinopathy, optic neuropathy, and neovascular glaucoma reported in up to 12% of cases (4, 13, 14). Moreover, EBRT typically requires daily treatment over long treatment periods of 3–5 weeks, which can be burdensome for patients with advanced systemic disease (13). For patients with better general condition or longer expected survival, more advanced and focalized radiation techniques such as plaque brachytherapy or GKR should be offered. Plaque brachytherapy has been considered the gold standard in localized radiation treatment, as mentioned in several studies (15, 16), which involves the surgical placement of a radioactive plaque on the sclera to allow direct radiation to the tumor while minimizing exposure to the adjacent tissues (4). This method achieves local tumor regression in up to 94%–100% of cases, with relatively good visual prognosis (3, 7, 11, 17, 18). However, its disadvantages, such as its invasive nature, the need for general anesthesia, the requirement for two separate surgical procedures (for plaque insertion and removal), the limited intraoperative accessibility to tumors posterior to the equator and those close to the optic nerve, limit its applicability especially in patients with poor systemic health (4, 15). The rates of complications, including radiation retinopathy, papillopathy, and cataract in anteriorly placed plaques, have been reported to be around 8% (7, 13, 18).

Gamma knife stereotactic radiotherapy (GKR) uses multiple converging beams of ionizing radiation to deliver highly focused radiation to the target lesion while preserving the surrounding healthy tissues. Originally developed for intracranial lesions, it has been adapted to treat intraocular tumors, particularly those not amenable to brachytherapy due to the tumor size, the posterior location, or the proximity to critical structures such as the optic nerve (15, 19). Its up to submillimeter precision allows it to target small or deeply seated lesions and to achieve effective results with lower overall radiation doses (typically around 30 Gy for choroidal metastasis), thereby reducing the risk of collateral damage and dose-related side effects such as radiation retinopathy, which is more frequently observed at cumulative doses exceeding 45 Gy (15). Unlike brachytherapy, GKR is less invasive and often requires only a single outpatient session. This minimizes hospital stays and procedural risks (7). Furthermore, it allows for the simultaneous treatment of multiple metastatic sites, as demonstrated in our case involving concurrent brain and ocular metastases, thereby offering a less physically taxing option compared with the prolonged and fractionated dosing of EBRT (7, 15, 22, 23).

Since its advent, GKR has been used as an efficacious tool in regressing choroidal melanomas. Parker et al. (14), in a systematic review of 52 studies and a pooled meta-analysis of 28 studies, demonstrated local control rates of uveal melanomas and choroidal metastases with GKR comparable to those achieved with plaque brachytherapy (90% in the COMS trial) and superior in some comparisons to EBRT (3, 10–12, 15, 17, 18). Notably, Georgopoulos et al. reported comparable control rates between GKR and brachytherapy (100% *vs*. 98% at 2 years) (15). Wackernagel et al., an Austrian study group that treated 189 patients with GKR for choroidal melanomas, achieved local tumor control in 94.4% of the patients. The estimated tumor control rates were 97.6% at 1 year, 94.2% at 5 years, and 92.4% at 10 years after treatment (19). However, very few cases focused on its use in the treatment of tumors metastasized to the choroid, less so in breast metastasis.

**Table 2** provides a summary of the cases reporting on the use of GKR specifically in choroidal metastasis. All reviewed studies, including ours, demonstrated excellent tumor control, with regression in all cases (7, 20–23). One patient with an underlying renal cell carcinoma was reported to have new choroidal metastasis post-complete regression of the original tumor, while another with an underlying non-small cell lung cancer had initial complete regression of the tumor followed by recurrence at 5 months (7). In our patient, GKR achieved anatomical tumor regression and complete resolution of retinal detachment, corroborating its efficacy in local tumor control comparable with other studies using higher doses. Our delivered dose was at the lower-to-mid range in comparison, highlighting the potential efficacy of a more conservative dosing in the treatment of similar cases using GKR.

**Table 2:** Summary of case studies reporting on the use of gamma knife radiosurgery (GKR) in choroidal metastasis.

Study/case	No. of patients	Age, gender	Primary tumor	Lesion location	GKR dose	BCVA pre-/final	Tumor response	Brain metastasis	Side effects	Follow-up (months)
Marchini et al. (20)	1	48, M	Lung	Temporal/inferior posterior pole	25 Gy at 50% isodose	0.6 LogMAR → 0.2 LogMAR	Tumor reduced to <1.5 mm at 3 months	No	Not reported	6
Lally et al. (21)	1	Late 40s, F	Breast	Juxtapapillary along the inferior arcade	18 Gy at 50% isodose	6/6 → 6/6	Complete regression	No	Early retinopathy (at 1 year)	12
Ares et al. (22)	3	62–74, 2M 1F	Melanoma, thyroid, lung	1 at the retinal periphery and ciliary body; 2 at the choroid	14–20 Gy at 50%–55% isodose	6/120 → 6/32, 6/12 → 6/30, 6/6 → 6/6	Regression of all tumors (1 complete regression)	All 3	Exudative retinopathy (1)	4–15
Cho et al. (7)	7	51–71, 4M 3F	Lung, gastric, renal	1 at the inferior choroid; others not reported	15–25 Gy at 50% isodose	Varied (LogMAR): 0.15 → 0.15, NPL → NPL, HM → HM, 0.08 → 0.8, 1.0 → 1.0, 0.2 → 0.8, 0.9 → 0.2	5 regression, 1 recurrence, 1 new metastasis	4	Worsened cataract (1), eye pain (1)	Mean, 8 months
Oliverio et al. (23)	1	37, F	Breast	Inferotemporal retina extended to the ora serrata	30 Gy (given in 10 days)	20/63 → 20/32	Tumor reduced to 2.88 mm, RD resolved	Yes	No	6
Our case	1	44, F	Breast	Papillomacular bundle with foveal involvement	16–18 Gy at 50%–90% isodose	CF → CF	Complete regression	Yes	Foveal atrophy	6

*M*, male; *F*, female; *BCVA*, best-corrected visual acuity; *LogMAR*, logarithm of the minimum angle of resolution; *NPL*, no perception of light; *CF*, counting fingers; *RD*, retinal detachment.

The visual outcomes, on the contrary, were inconsistent across studies. While some cases experienced significant BCVA improvement, others remained stable or deteriorated. Our case demonstrated an initial BCVA improvement to 6/18 at 2 months post-treatment, however later deteriorated to CF by 6 months despite anatomical improvement. This poor visual outcome is likely due to foveal atrophy, as evident on the OCT findings, potentially caused by either previous disease activity, prolonged photoreceptor–RPE separation, or radiation-induced retinal damage. Unlike other commonly reported causes of post-GKR visual decline such as cataract progression, elevated IOP, glaucoma, and radiation retinopathy (7), foveal atrophy is rarely documented elsewhere. In our patient, its development was likely due to the proximity of the tumor to the fovea and the papillomacular bundle. Visual deterioration following GKR has also been associated with baseline visual function, tumor size and location, and the degree of retinal damage, with worse initial visual acuity predictive of poorer vision post-treatment (15). With this in mind, careful patient selection and thorough counseling of patients regarding vision expectations are important before subjecting patients to GKR.

This pattern of delayed visual acuity deterioration has also been observed in previous studies, with Parker et al. reporting that only 27% of patients had stable or improved vision post-GKR, dropping to 19% when short follow-up periods of less than 8 months were excluded (15). In fact, the study suggests that complications and vision deterioration may take 6–8 months to manifest; hence, case reports with shorter follow-up durations may underestimate the true complication rates. These findings underscore the importance of recognizing that GKR-related complications and visual decline may emerge months after treatment.

Importantly, the procedure was well tolerated by our patient. The procedure lasted under 60 min, and the patient experienced no discomfort, inflammation, complications, or ocular surface disease both intraoperatively and postoperatively. To the best of our knowledge, there are no established guidelines outlining the optimal technique for GKR in the treatment of choroidal metastasis. Eye globe immobilization during the procedure is crucial to ensure accurate radiation delivery. In our patient, this was achieved via retrobulbar anesthesia combined with external fixation of the extraocular muscles (EOM). Temporary tarsorrhaphy was performed to avoid corneal exposure. Throughout the entire procedure, there was no ipsilateral eye movement, which allowed for a smooth and efficient delivery of the gamma knife. This case also demonstrated the successful use of GKR in simultaneously regressing both choroidal and brain metastases under one setting, offering a physically less draining alternative for the patient. Furthermore, it is also a mean to spare the patient from painful sequelae of the choroidal tumor, such as neovascular glaucoma and potential evisceration. Our patient was discharged the following day, although the literature has reported performing GKR as a same-day outpatient procedure without complications (14, 22). With the short treatment duration, we were able to reduce healthcare issues with surgical admissions and bed occupancy and minimize the risk of hospital-related complications. Hence, our experience with GKR reflected the logistical ease and patient comfort with this treatment modality.

Over recent years, CyberKnife radiosurgery has also emerged as a frameless alternative for stereotactic treatment of choroidal metastasis. Similarly to GKR, it delivers a highly conformal, submillimeter-precision radiation to tumors, with reported complete or almost complete tumor regression, minimal ocular toxicity, and variable visual outcomes (24). Both approaches enable the simultaneous targeting of multiple lesions and rapid outpatient treatment. Their primary distinction lies in their immobilization: while GKR secures using a rigid stereotactic head frame and fixed cobalt-60 sources, CyberKnife uses real-time image guidance and robotic beam adjustments to compensate for motion during delivery (24). Naturally, this frameless approach improves comfort and is preferable for patients who cannot tolerate invasive frame fixation. Comparative intrafraction motion studies have found slightly greater movement with frameless-based systems compared with rigid frames (25). Although modern image-guided correction protocols have largely reduced this discrepancy to clinically acceptable levels (25, 26), many centers might still favor frame-based immobilization for very small posterior lesions near critical visual structures, given its extensive validated outcome data and minimal geometric uncertainty. The available evidence for CyberKnife in choroidal metastasis remains limited to small series and isolated case reports, with scarce data on its use in the concurrent treatment of ocular and brain metastases. Comparative studies between the two modalities are lacking and would be of clinical benefit.

This paper also underscored the importance of a multidisciplinary care model, involving the ophthalmology, neurosurgery, surgical, and oncology teams. Coordinated management is critical in the treatment of complex cases such as metastatic cancer, where multiple organ systems are involved. The successful use of GKR in this case reflects the collaborative effort across specialties in contributing to a more comprehensive treatment plan.

## Conclusion

In conclusion, GKR is an effective, noninvasive alternative for the management of choroidal metastasis, particularly in cases where conventional treatments such as plaque brachytherapy or EBRT are unsuitable. While our case demonstrates excellent tumor regression and resolution of retinal detachment with GKR, the long-term visual outcomes are suboptimal due to foveal atrophy likely secondary to prior disease activity or radiation treatment and are largely dependent on the tumor location. Our case has also reinforced its value in the management of complex metastatic scenarios involving both choroidal and brain lesions while underscoring the need for ongoing visual monitoring. Further studies with larger cohorts and longer follow-up periods are needed to fully define the role of GKR in ocular metastasis and to establish standardized protocols for its use.

## Data Availability

The original contributions presented in the study are included in the article/Supplementary Material. Further inquiries can be directed to the corresponding author.
